# Localized Intra-Cavitary Therapy to Drive Systemic Anti-Tumor Immunity

**DOI:** 10.3389/fimmu.2022.846235

**Published:** 2022-02-11

**Authors:** Vera S. Donnenberg, Patrick L. Wagner, James D. Luketich, David L. Bartlett, Albert D. Donnenberg

**Affiliations:** ^1^ University of Pittsburgh Medical Center (UPMC) Hillman Cancer Centers, Pittsburgh, PA, United States; ^2^ McGowan Institute for Regenerative Medicine, Pittsburgh, PA, United States; ^3^ Department of Cardiothoracic Surgery, University of Pittsburgh School of Medicine, Pittsburgh, PA, United States; ^4^ Surgical Oncology, Allegheny Health Network Cancer Institute, Pittsburgh, PA, United States; ^5^ College of Medicine, Drexel University, Pittsburgh, PA, United States; ^6^ Department of Medicine, University of Pittsburgh School of Medicine, Pittsburgh, PA, United States

**Keywords:** malignant pleural effusion, malignant ascites, local therapy, intratumoral, epithelial to mesenchymal transition (EMT), video assisted thoracoscopic surgery (VATS)

## Abstract

Metastasis to the pleural and peritoneal cavities is a common terminal pathway for a wide variety of cancers. This article explores how these unique environments both promote aggressive tumor behavior and suppresses anti-tumor immunity, and ways in which local delivery of protein therapeutics can leverage the contained nature of these spaces to a therapeutic advantage, achieving high intra-cavital concentrations while minimizing systemic toxicity.

## Introduction

Malignant pleural effusions (MPE) have a US incidence of more than 150,000 cases per year (1, 2) and a life expectancy measured in months (3). Likewise, patients with malignant peritoneal ascites (MPA) and peritoneal carcinomatosis also have abysmal survival (4) and poor quality of life (QOL (5),) with patients experiencing multiple hospitalizations for bowel obstruction and pain (6). Both MPE and MPA are common, painful, difficult to treat, and most importantly are uniformly fatal. Despite significant clinical progress in immuno-oncology, there has been almost no change in survival or quality of life for patients with MPE or MPA, which become the proximate cause of death in many cases of advanced cancer

## Subsections

### An Incomplete Understanding of the Pleural and Peritoneal Cavity Environments

**Figure 1:**
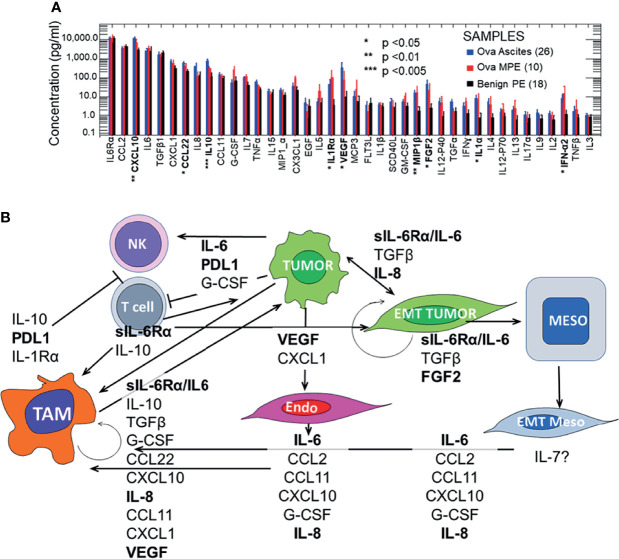
Pleural and peritoneal secretomes in ovarian cancer and benign pleural effusions. Panel **(A)** Pleural and peritoneal secretomes are dominated by immunosuppressive and EMT tumor promoting cytokines and chemokines (IL-6/IL-6Rα, VEGF, IL-8, CXCL10, and IL-10). Data were log-transformed for analysis. There were no significant differences between analytes in ovarian ascites and malignant pleural effusions (MPE). Data for ovarian ascites and MPE were pooled and compared to Benign PE (Student’s 2-tailed t test). Results were Bonferroni corrected for 40 comparisons. Statistically significant comparisons are indicated in bold typeface. Effusions and ascites were collected as anonymized medical waste under a University of Pittsburgh IRB exemptions (Nos. 0503126 and 0403111). Thirteen pleural effusions were collected from patients without pleural malignancy (11 with heart failure, 2 with asbestosis). The benign effusion data have been published previously (7, 8). Cytokines were quantified on the Luminex platform, using the Curiox LT-MX plate washer, Curiox DA-96 plates, the Luminex 200 System analyzer and xPonent data acquisition and analysis software. Cytokines were measured in 5 µL of neat, clarified effusion using the MILLIPLEX MAP Human Cytokine/Chemokine Magnetic Bead Panel - Premixed 38 Plex (Cat. No. HCYTMAG-60K-PX38), MILLIPLEX MAP Human TGFβ (Cat. No. TGFBMAG-64K-01) and IL-6Rα from the Human Angiogenesis/Growth Factor Panel 2 (Cat. No. HANG2MAG-12K-01) kits, as previously published (Donnenberg et al., https://doi.org/10.18632/oncotarget.27290). Panel **(B)** Effects of the malignant secretome on cavitary cell types and mesothelial maintenance. The 10 most prominent cytokines and chemokines (geometric mean ≥ 100 pg/mL) in intracavitary ovarian cancer are shown, plus PDL1 which is expressed on intracavitary macrophages and must be addressed for successful intra-cavitary therapy. The variety of cell types, cytokines and chemokines involved, and the potential to amplify effects through autocrine and juxtacrine feedback loops justifies the need for multimodal intra-cavitary therapy. Potential therapeutic targets for which on- or off-label FDA approved agents are currently available are shown in bold typeface.

In states of normal health, the pleural and peritoneal cavities contain physiologic fluid with a dynamic array of immune cells and unique secretomes. In non-neoplastic pathologic states, fluid can accumulate in these cavities due either to transudative mechanisms (vascular pressure and decreased resorption), or exudative mechanisms (tissue inflammation and immune cell infiltration). These processes result in dramatic changes to the cavitary secretome and immune environment. Malignant effusions and ascites also exhibit similar shifts in the secretome and cellular infiltrate, but several additional cytokines and chemokines distinguish them from benign effusions (**Figure 1A**). The presence of additional malignancy-specific components notwithstanding, the combination of cytokines seen across the spectrum of benign and malignant conditions is predicted to drive both aggressive tumor behavior and polarize pleural immune cells away from tumor-specific immunity and instead, toward a maladaptive repair-and-regenerate mode. Understanding the interplay between the secretome and the resident cell populations will provide much needed foundation for the development of innovative immunotherapeutics targetable to the cavitary spaces and specific to the mechanisms relevant to malignant effusions and ascites.

### The Secretomic Signatures of Malignant Peritoneal Ascites and Pleural Effusions Are Indistinguishable

One of the most striking features of **Figure 1A**, which contrasts the secretomes of ovarian cancer ascites and pleural effusions with that of benign pleural effusions, is the similarity between the cytokine and chemokine profiles in pleural and peritoneal malignancies. In the healthy cavitary spaces, a physiologic secretome and a complement of immune cells are dedicated to maintenance and repair of the mesothelial lining through a process involving the epithelial to mesenchymal transition (EMT, detachment, migration) and the mesenchymal to epithelial transition (MET, reattachment, re-epithelialization) (9). In malignant fluid, the environment is transformed into an inflammatory milieu that promotes wound healing and suppresses adaptive immune effector responses. As mentioned above, this tumor-related cavitary fluid environment has all the inflammatory components of benign effusions (IL-6, sIL-6Rα, CCL2, CXCL10, TGFβ, CCL22, IL-8, IL-5 **Figure 1**), but includes additional tumor and endothelial growth factors, as well as a blunted effector cytokine response (IFNα2) that is not seen in physiologic cavitary fluid or benign effusions.

### Effects of the Malignant Secretome on Mesothelial Maintenance, Intra-Cavitary Tumor and Anti-Tumor Responses

Because the pleural and peritoneal spaces are lined by mesothelial cells, and because effusions and ascites result from a net inflow from the peripheral circulation, these cavities develop a unique cytokine environment. In the context of malignancy, the milieu of cytokines is predicted to play a maladaptive, tumor-supporting role, with negative effects not only in the cavitary environment, but systemically as well, promoting motility and solid organ metastasis. **Figure 1B** illustrates mechanisms by which the inflammatory cavitary secretome, initiated by tumor metastasis and supplemented by tumor-secreted cytokines, is predicted to establish an environment that promotes aggressive tumor behavior. These mechanisms include EMT, suppression of adaptive T-cell effector responses, and polarization of macrophages to support rather than oppose tumor growth. Although ovarian cancer is presented here as an example, the malignant cavitary secretome, and the resulting tumor pathobiology is common to a wide range of cancers (7, 8).

### A Rationale for Intra-Cavitary Therapy Using Immune-Oncology Drugs

The physiologically isolated pleural and peritoneal environments provide ideal anatomical spaces for the localized administration of large protein drugs. The pleura as well as the peritoneum are lined with mesothelial cells joined by tight junctions (10) creating a unique and isolated cavitary environment. Unlike chemotherapeutics, high molecular weight immuno-oncology drugs remain concentrated when administered directly to these cavities, reaching a high target occupancy even with protein drugs with a narrow therapeutic index when administered systemically (11–14).

Local administration of therapeutics may be used to directly target the tumor, support local immune cells, and condition cancer associated stromal cells (15). Despite the net inflow of serous fluid, IgG levels are lower in pleural effusions (16) and peritoneal ascites than in the peripheral circulation. Thus, intravenous administration of protein therapeutics may not be the most effective way to achieve the necessary therapeutic levels within the cavitary spaces. Conversely, localized intra-cavitary administration of these therapeutics has been shown to result in low systemic exposure, and negligible on-target off-tumor effects, while reducing adverse events associated with systemic toxicity (13, 14). The same principle applies to intra-cavitary injection of RNA-based therapeutics (17, 18). This mode of administration is greatly facilitated by the use of minimally invasive surgical techniques and placement of indwelling catheters. While these catheters are traditionally used for palliative decompression of the cavitary spaces, they have been repurposed as drug delivery devices, for example the instillation of pleurodesis agents or cytotoxic chemotherapy. Video assisted thoracoscopic surgery [VATS (19)] or laparoscopic peritoneal catheter placement (20) can likewise be used to guide intracavital-intratumoral drug delivery for agents such as oncovaccines and mRNA therapeutics, which are injected directly into tumor foci. Further, indwelling catheters allow for iterative sampling of cavitary fluid to monitor the response to therapy, the impact on the cavitary immune environment, the pharmacokinetics and pharmacodynamics (PK/PD) of personalized drug dosing, and the real time determination of minimal anticipated biological effect levels [MABEL (21)]. Finally, anti-tumor effector responses initiated in the confines of the cavitary spaces would be expected to propagate systemically through the draining lymphatics, where they could combat solid organ metastases. Although most studies of single-agent intracavital immunotherapies have not measured effects on systemic immunity directly, the intrapleural (14) and intraperitoneal (13) experience with the bispecific anti-CD3/anti-EpCAM antibody catumaxomab is informative. Even though the systemic catumaxomab concentration was <1% of the intracavitary concentration, both studies observed transient increases in serum transaminases, which were attributed to systemic cytokine release. Similarly, intracavitary IL-2 administration resulted in an increase in peripheral CD8 T cells expressing granzyme B (22) and in peripheral NK cell activity (12), and intracavitary administration of an adenovirus/interferon β construct resulted in increased peripheral NK activity and anti-tumor antibodies (23).

In this manner, the cavitary spaces can be conceived of as ‘bioreactors’ into which novel immunotherapeutic agents could be instilled or injected intratumorally with the aim of selecting and stimulating effector cytotoxic T cells for cavitary and abscopal activity. We argue for local rather than systemic administration of protein-based therapies, since systemic administration may not achieve the necessary intracavitary therapeutic levels (11).

Intra-cavitary therapy can be considered as a special case of intra-tumoral therapy (reviewed in (24). Intra-tumoral immunotherapies have been explored extensively with the intention of altering the immune microenvironment of solid tumors to promote adaptive immunity. The peritoneal and pleural cavities are fluid-phase environments that may be more amenable to targeted manipulation. Although clusters of tumor cells are usually demonstrable in the fluid phase, the bulk of the lesions cake the mesothelium and adhere to internal organs, where they are bathed in the fluid phase.

### Effusions and Ascites as a Source for Adoptive Cellular Therapy

Adoptive T-cell therapy using autologous tumor infiltrating lymphocytes (TIL) has been reported to induce salvage responses in a variety of refractory solid tumors (25). Conventionally, TIL therapy requires large-scale expansion of a small number of T-cells grown out from tumor tissue fragments stimulated with high dose IL-2 and anti-CD3 antibody. Since the expanded TIL depend on the continued presence of IL-2 for their survival, TIL infusion must be accompanied by repeated systemic administration of high dose IL-2, stopping only when dose-limiting toxicity is reached. TIL infusion is often preceded by treatment with immunosuppressive chemotherapeutic agents such as cyclophosphamide and fludarabine to make space for the therapeutic cells. Therapeutic drainage of MPE frequently yields on the order of 0.25 to 0.5 x 10^6^ pleural T cells/mL. In our experience, it is not unusual to drain a liter of fluid, yielding up to 5 x 10^8^ T cells. Macrophages are also prevalent, constituting up to 50% of total nucleated MPE cells. Thus, in a single drainage it is often possible to obtain potentially therapeutic doses of pleural T cells following short-term activation or expansion. The *ex vivo* activated cells can be positively selected for CD45+ T cells and macrophages and then re-instilled into the pleural or peritoneal cavity. Potential advantages over conventionally expanded TIL include greatly simplified and rapid manufacture, potentially eliminating the requirement for systemic administration of toxic high dose IL-2 to ensure cell survival after reintroduction.

### Potential Drawbacks to Intra-Cavitary Therapeutics

The principal problems facing intra-cavitary therapies are determining the most efficacious combination of therapeutics and the logistics associated with intra-cavitary and intratumoral administration. Additionally, the high cost of biologics, and particularly patient-specific cellular therapeutics must also be considered. There are a broad range of potential modalities that can be locally delivered, and within these modalities a variety of agents with similar targets or mechanisms of action, with many more in pre-clinical or early phase clinical trials (24, 26, 27). The varied formulations and compatibilities of potentially useful therapeutics must be considered if they are to be co-administered through indwelling catheters. Some therapeutics will be administered into the cavities, but others will require precise intratumoral delivery, which is limited to accessible tumor. Drug retention at intratumoral injection sites poses an additional potential difficulty and must be addressed by choosing appropriate agents and vehicles. For example, intratumoral injection of liposomal IL-12 mRNA (28) is more likely to remain localized than injection of the cytokine itself. Unexpected toxicities resulting from localized immune hyperresponsiveness, or interference with normal tissue maintenance may also pose problems, especially if they are delayed. Quantification of responses will likely require objective criteria similar to the RECIST score for solid tumors (29).

Technical challenges to implementation of intra-cavitary therapy include the need for dedicated personnel and facilities, including those required for image guided drug delivery. For drug delivery protocols requiring general anesthesia, the ability to administer repeated doses will be limited. Toxicities specific to intraperitoneal immunotherapy may be anticipated based on the experience with intra-peritoneal IL-2 (30) and monoclonal antibody (31) therapy (pyrexia, abdominal pain, nausea/vomiting). These toxicities may be cavity specific as they were far milder with intrapleural administration of the same cytokine (12, 32) or antibody (14).

Finally, maximizing benefit with respect to cost is a challenge that must be met if intracavitary therapy is to gain acceptance. Given the dire prognosis and current palliative approaches to cavitary malignancies, any therapeutic combination that can provide an objective increase in response rates and survival with improved quality of life may justify the current high cost of immunotherapeutics. However, once Phase I/II trials have been completed, it will be important to initiate therapy while patients still have acceptable performance status and limited disease burden (33).

## Discussion

**Figure 2:**
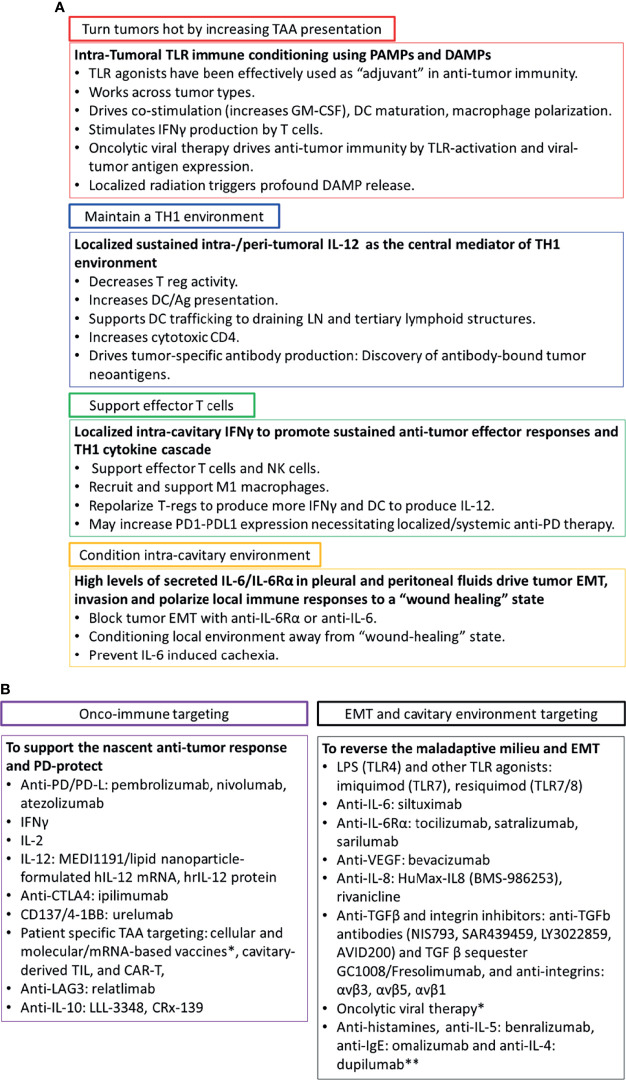
Intra-cavitary therapeutics to drive systemic immunity and reverse EMT. Panel **(A)** Since tumors that metastasize to the pleural and peritoneum exist in an environment tailored to EMT and immune suppression, combination therapy directed toward conditioning the local environment as well as activating anti-tumor immunity is required. Panel **(B)** A list of potential therapeutics directed toward reversing tumor EMT, repolarizing the cavitary maladaptive milieu, and driving local and systemic anti-tumor immunity. *Summarized in Addeo et al. (21) **Targeting histamine and related cytokines. Discussed in Li et al. (34).

Since tumors that metastasize to the pleura and peritoneum exist in an environment tailored to EMT and immune suppression, combination therapy directed toward conditioning the local environment as well as activating anti-tumor immunity is warranted. **Figure 2A** divides these goals into four categories that can be addressed with intra-cavitary and intratumoral therapies: 1) Turning *cold* tumors *hot*; 2) Increasing tumor-associated antigen presentation; 3) Supporting effector T cell responses; and 4) Conditioning the local environment to block EMT.

Tumors can be made more immunogenic by inducing damage associated molecular patterns (DAMPS) with therapies such as radiation, or pathogen associated molecular patterns (PAMPS) *via* oncolytic virus therapy (35). Tumors may also constitutively express receptors for PAMPS and DAMPs (toll-like receptors, TLR), but their prognostic significance varies with disease and receptor type (36). Introduction of TLR ligands through natural infection of the pleura (empyema) has been associated with prolonged survival in patients with cancer metastatic to the pleura or lung cancer (37, 38). This response may be due to PAMP-associated repolarization of the local immune environment, with concomitant alternation of the cytokine profile and augmentation of tumor antigen presentation by resident macrophages and dendritic cells. Numerous attempts have been made to exploit TLR receptor agonists as single agent therapeutics with limited success. This does not rule out the possibility that they will be a highly effective adjuvant to other immune oncology interventions.

IL-12 plays a central role in inducing and maintaining Th1 T-cell polarization (39). The low levels of IL-12p70 in pleural and ascites fluid (**Figure 1A**) suggest that IL-12 can be a key therapeutic for conditioning the intra-cavital environment. Multiple clinical trials of IL-12 protein and IL-12 mRNA support its potential use in intra-cavitary therapy, where systemic adverse effects can be limited. IFNγ promotes T-cell and NK effector cells both directly and indirectly. Like IL-12, local administration may be advantageous to achieve functional concentrations while limiting systemic toxicity. Finally, therapeutic T cells may be activated or expanded *ex vivo* for intra-cavitary administration, provided that they are not reintroduced into an immunosuppressive environment.

Our secretomic data supports the role of IL-6/sIL-6Rα receptor trans-signaling as the key driver of tumor EMT and associated therapy resistance and increased metastatic potential. IL-6 and its secreted receptor sIL-6Rα are increasingly recognized as master cytokines (40, 41), upstream of a wide array of inflammatory processes, including pathologies as diverse as cytokine release syndrome (42), acute allograft rejection (43), rheumatoid arthritis (44), asbestosis (45) and cachexia (46). Complexes of soluble IL-6/IL-6Rα elicit responses from gp130-expressing cells that lack the complete IL-6 receptor by a process known as trans-signaling (40, 47). **Figure 1A** and our findings in NSCLC-associated MPE (7) reveal a profound degree of cytokine-chemokine polarization dominated by ng/mL concentrations of IL-6/sIL6Rα. Neutralizing pleural IL-6 or IL-6Rα activity with therapeutic antibodies may not only diminish IL-6-driven aggressive tumor behavior associated with EMT (48) (**Figure 1B**), but may also reverse downstream negative regulation of tumor-specific immune effector responses, thereby enhancing the efficacy of other immune oncology therapies. Although long-term antagonism of IL-6Rα is immunosuppressive (49), single dose exposure has been shown to break the cytokine storm associated with CAR-T therapy without compromising effector responses (50) or incurring serious adverse events (51). Intra-cavitary administration of anti-IL-6 or anti-IL-6Rα may likewise be expected to exert profound effects on the pleural or peritoneal environments.

Histamine has been shown to play a role in conferring resistance to immunotherapy. H1 antihistamine therapy counteracts histamine-mediated immunosuppression by counteracting M2 macrophage polarization and promoting CD8+ effector T cell responses (34). In the intracavitary space, cytokines such as IL-4 and IL-5 that are elevated in MPE (**Figure 1**) and are central to the allergy cascade (52) and may provide additional targets of therapy.

The pleura and peritoneum are common sites of metastasis for a wide variety of cancers. Their unique physiology makes them ideal tumor sanctuaries that promote aggressive behavior while inhibiting immune effector responses. The contained nature of these spaces also presents an opportunity to therapeutically manipulate the tumor environment in ways that are not possible for other metastatic lesions. There is a wealth of agents available for combination intra-cavitary therapy (**Figure 2B**), many of which have already shown some activity as single agents. We argue that combination therapies designed to condition the maladaptive cavitary environment, reverse EMT and stimulate immune priming locally and systemically will succeed where single agents have ultimately failed. However, the devil is in the details, and the challenge will be to design and implement the most agile adaptive therapeutic trials (53) designed to determine the safest and most effective therapeutic combinations and dosing schedules.

## Data Availability Statement

The raw data supporting the conclusions of this article will be made available by the authors, without undue reservation.

## Author Contributions

AD and VD wrote the manuscript, created and analyzed the data. PW contributed the section on peritoneal malignancies and edited the manuscript. JL provided samples and contributed to the discussion and ideas. DB contributed to the discussion and ideas. All authors contributed to the article and approved the submitted version.

## Funding

This work was supported by BC032981, BC044784, W81XWH-12-1-0415 and BC132245_W81XWH-14-0258 from the Department of Defense, National Cancer Institute (NCI) grant R21 CA191647, MetaVivor FP00002718, the Glimmer of Hope Foundation, the Pennsylvania Breast Cancer Coalition, and the David Downing Fund. The Hillman Cancer Center Luminex Facility is supported by Cancer Center Support Grant P30CA047904.

## Conflict of Interest

The authors declare that the research was conducted in the absence of any commercial or financial relationships that could be construed as a potential conflict of interest.

## Publisher’s Note

All claims expressed in this article are solely those of the authors and do not necessarily represent those of their affiliated organizations, or those of the publisher, the editors and the reviewers. Any product that may be evaluated in this article, or claim that may be made by its manufacturer, is not guaranteed or endorsed by the publisher.
